# Automated synthesis of [^68^Ga]Ga-FAPI-46 without pre-purification of the generator eluate on three common synthesis modules and two generator types

**DOI:** 10.1186/s41181-022-00172-1

**Published:** 2022-07-29

**Authors:** Ammar Alfteimi, Ulf Lützen, Alexander Helm, Michael Jüptner, Marlies Marx, Yi Zhao, Maaz Zuhayra

**Affiliations:** Department of Nuclear Medicine, Molecular Diagnostic Imaging and Therapy, University Hospital of Schleswig-Holstein (UKSH), Campus Kiel, Karl Lennert Cancer Center North, Feld-Str. 21 (Haus L), 24105 Kiel, Germany

**Keywords:** Automated radiosynthesis, FAPI-46, Fibroblast, Gallium-68

## Abstract

**Background:**

The recent development of quinoline-based radiotracers, which act as fibroblast activation protein inhibitors (FAPIs), has shown promising preclinical and clinical advantages. [^68^Ga]Ga-FAPI-46 is a new radiotracer for in vivo detection of the fibroblast activation protein by positron emission tomography (PET). Recently, the automated synthesis of [^68^Ga]Ga-FAPI-46 was reported based on pre-concentration and purification of the generator eluate by using a cation exchange-cartridge.

Our aim was to simplify the synthesis and shorten the automated synthesis of [^68^Ga]Ga-FAPI-46 to make it accessible and thus even more attractive to a broader clinical and scientific community.

**Results:**

We developed and evaluated the GMP compliant automatic synthesis of [^68^Ga]Ga-FAPI-46 using two different ^68^Ge/^68^Ga generators (an Eckert & Ziegler, GalliaPharm generator, 1.85 GBq/50 mCi and an iThemba generator, 1.85 GBq/50 mCi) Somerset West, South Africa) and three different commercial and customized systems: the EasyOne module from Trasis; the GaSy module from Synthra with a customized synthesis template and a customized single use cassette. Additionally, the automatic synthesis of [^68^Ga]Ga-FAPI-46 was established on a GallElut synthesis module from Scintomics with fixed tubing.

**Conclusions:**

Independent of the synthesis modules or the generators employed we were able to complete the synthesis of [^68^Ga]Ga-FAPI-46 in 12 min including the process of purification and formulation. In all cases, the final products showed more than 99.5% chemical purity and the radiochemical yield reached around 92.5% (decay corrected). All quality control parameters (e.g. sterility, stability and radiochemical purity) were conform to the European Pharmacopoeia.

## Background

The fibroblast activation protein (FAP) is a membrane bound serine protease belonging to the dipeptidyl peptidase 4 (DPP4) family (Edosada et al. 2006). Interestingly, FAP is not expressed in normal healthy tissue but overexpressed by cancer-associated fibroblasts of several tumor entities (Brennen et al. 2012; Park et al. 1999; Kratochwil et al. 2019). However, FAP expression is detected in the stroma of more than 90% of human cancer (Wikberg et al. 2013). It plays an important role in a variety of tumor promoting activities such as chemotherapy resistance, angiogenesis, matrix remodeling, and immunosuppression and helps tumor cells invade surrounding tissue (Lindner et al. 2021). Therefore, it represents a potent biomarker for cancer diagnosis and prognosis and is considered as an important target for clinical diagnostic and therapeutic applications in nuclear medicine (Lindner et al. 2019; Puré and Lo 2016; Altmann et al. 2021; Jiang et al. 2016; Windisch et al. 2020).

**Fig. 1:**
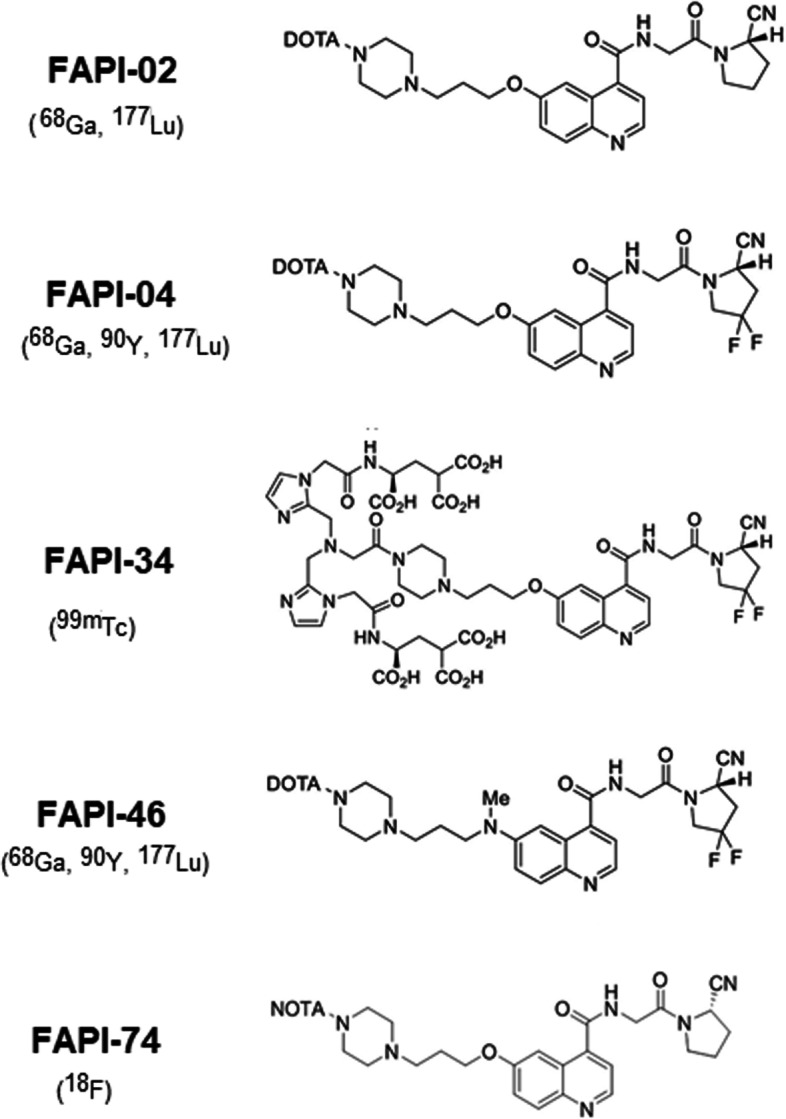
Chemical structures of FAPI precursors used in clinical application with nuclides in brackets (Lindner et al. 2019)

Quinoline-based radiotracers, which act as fibroblast activation protein inhibitors (FAPIs), have recently shown promising preclinical and clinical advantages (Syed et al. 2020). Therefore, a variety of FAP inhibitors (FAPI), such as FAPI-02, FAPI-04, FAPI-34, FAPI-46 and FAPI-74, have been developed for labeling with different nuclei according to the clinical purpose, such as ^68^ Ga,^18^F and ^177^Lu (Fig. 1) (Terry et al. 2015; van der Geest et al. 2018; Altmann et al. 2021).

The most promising molecule among those is FAPI-46, as several common cancers were identified by [^68^Ga]Ga-FAPI-46 PET/CT with remarkably high uptake and high image contrast (Kratochwil et al. 2019; Meyer et al. 2019). PET images showed a strong correlation between FAP expression und uptake of [^68^Ga]Ga-FAPI-46 (Sharma et al. 2021). The high and rather selective tumor uptake opens up new perspectives for non-invasive tumor characterization or therapy (Kratochwil et al. 2019).

[^68^Ga]Ga-FAPI-46 was synthesized first by Spreckelmeyer (Spreckelmeyer et al. 2020) using two different synthesis modules: Modular Lab PharmTracer (MLPT) and Modular Lab eazy (ML eazy) from Eckert & Ziegler Eurotope GmbH for clinical application. Their synthesis based on pre-concentration and pre-purification of the TiO_2_-generator eluate (GalliaPharm®, Eckert & Ziegler Radiopharma GmbH, Germany) using a cation exchange-cartridge. Recently, Da Pieve evaluated the fully automated synthesis of [^68^Ga]Ga-FAPI-46 for clinical applications on a Trasis AiO platform, which included the processing of the TiO_2_-generator eluate (Galli Ad, IRE ELiTE Radiopharma, Fleurus, Belgium) using a strong cation exchange resin and a final purification step through an hydrophilic-lipophilic balance (HLB) cartridge followed by a quaternary methyl ammonium (QMA) cartridge (Da Pieve et al. 2022). As in the case of Spreckelmeyer their synthesis of [^68^Ga]Ga-FAPI-46 also based on pre-concentration and pre-purification of the TiO_2_-generator eluate.

Currently most of the synthesis modules on the market, such as the Trasis, Scintomics or the Synthra synthesis module, have changed the routine synthesis of ^68^ Ga tracers to methods which do not need pre-purification steps in order to reduce the synthesis time. This is possible due to the improved properties of the generators, which now show a ^68^Ge breakthrough well below the European Pharmacopoeia limit of 0.001% (Tworowska et al. 2016).

The aim of our study was to demonstrate that GMP production of [^68^Ga]Ga-FAPI-46 could be achieved by using three common commercial and customized synthesis modules without need for pre-purification of the eluted [^68^Ga]GaCl_3_ from a SnO_2_- or TiO_2_-based ^68^Ge/^68^ Ga generator. Hereby, the automated synthesis of [^68^Ga]Ga-FAPI-46 was not only considerably simplified and accelerated but also maintained the high quality of the final product while maintaining high yields making [^68^Ga]Ga-FAPI-46 much more attractive for the use in clinical routine.

## Methods and materials

All single-use cassettes and reagent kits for the radio synthesis were sterile and manufactured under GMP (Trasis®, Ans, Belgium) and purchased from ABX (Radeberg, Germany), purchased from. Ascorbic acid (manufactured under GMP) was purchased from Avantor (Center Valley, PA). The FAPI-peptide was purchased in 50 µg single-use vials as a GMP product from SOFIE (Dulles, VA). Other chemicals were purchased from commercial sources and were used without further purification.

### Preparation of HEPES buffer solution (5 M, pH 5.7)

59.6 g of HEPES powder were put into a clean and sterilized bottle (100 ml) and 50 ml of ultrapure water were added to dissolve the powder. The bottle was closed with a sterile septum cap, then put in an ultrasonic water bath until the powder is dissolved completely. The pH was measured to be 5.7 ± 0.3. This buffer solution was filtered sterile and kept tightly sealed.

### Preparation of the hydrochloric acid solution (1 M)

90 ml of ultrapure water were filled into a sterile glass bottle. 10 ml of high-purity 30% hydrochloric acid solution were added and carefully mixed.

### Synthesis of [^68^Ga]Ga-FAPI-46 using a trasis easy one synthesizer

**Fig. 2:**
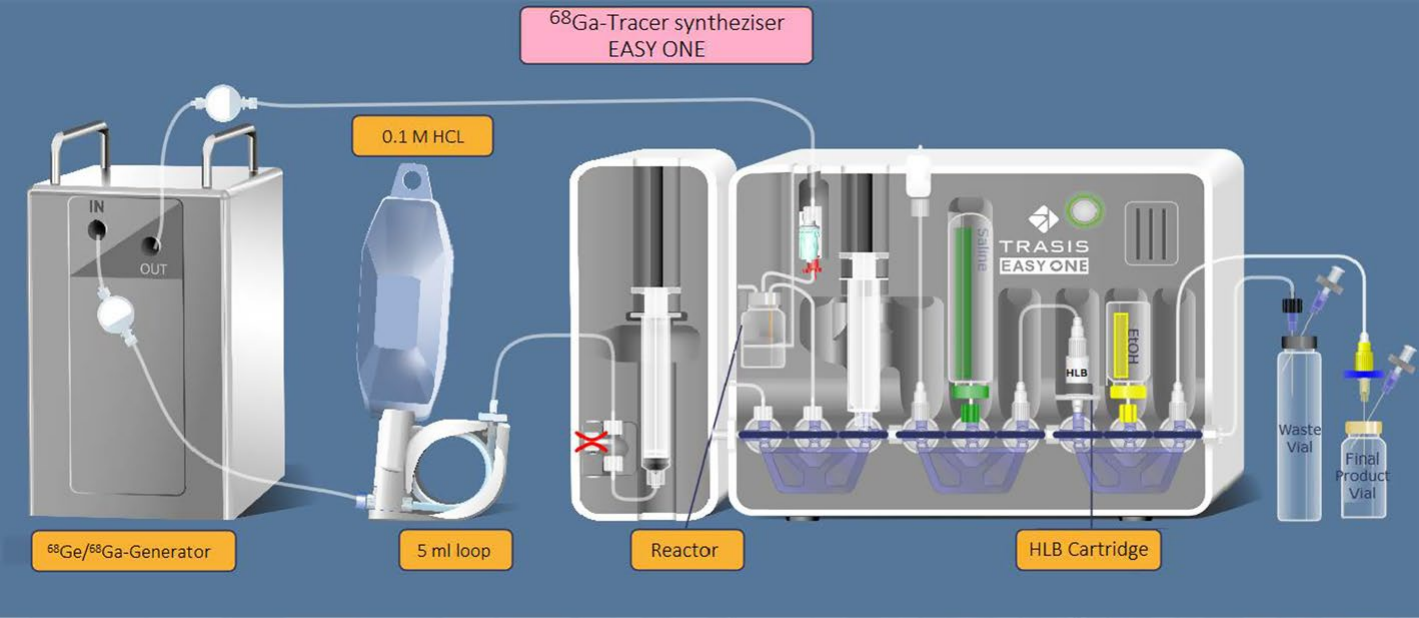
Automated synthesizer of [^68^Ga]Ga-FAPI-46 (EasyOne; Trasis)

For the fully automated synthesis of [^68^Ga]Ga-FAPI-46 employing a Trasis EasyOne synthesizer (operated by the software Trasis Supervision® (Trasis, Ans, Belgium)) shown in Fig. 2 we used the single-use cassette and reagent kit for the synthesis of ^68^Ga peptides supplied by Trasis (Trasis, Ans, Belgium). The reagent kit contains an acetate buffer syringe as well as saline and ethanol vials. The disposable cassette includes a 10 ml reactor, one 10 ml syringe and spikes for connection with two prefilled vials as well as an Oasis HLB cartridge. The reaction mixture containing 2 ml of acetate buffer solution (0.4 M, pH = 9.18), 0.3 mg ascorbic acid and 50 μg of FAPI-46 was loaded into the reaction vial.

[^68^Ga]GaCl_3_ was eluted from a ^68^Ge/^68^ Ga generator (Eckert & Ziegler, GalliaPharm generator, 1.85 GBq/50 mCi) with 5 ml of 0.1 M HCl. The labelling process involved preheating the reaction vessel containing the reaction mixture (90 °C), adding the [^68^Ga]GaCl_3_ solution and continued heating for 4 min.

For purification of the crude reaction mixture, the reaction mixture was passed through a HLB Sep-Pak cartridge to trap the labelled peptide by retaining the [^68^Ga]Ga-FAPI-46 while ^68^ Ga impurities passed through the HLB Sep-Pak cartridge into a waste vial. The HLB Sep-Pak cartridge was then washed with 5 ml of 0.9% aq. NaCl to remove most of the possibly remaining free [^68^Ga]GaCl_3_ as well as buffer and traces of ^68^Ge breakthrough. Then the [^68^Ga]Ga-FAPI-46 was eluted with 0.9 ml of ethanol, passed through a 0.22 μm filter and collected in the product vial before being further diluted with 9.1 ml of 0.9% aq. NaCl.

### Synthesis of [^68^Ga]Ga-FAPI-46 using a Synthra synthesizer

**Fig. 3:**
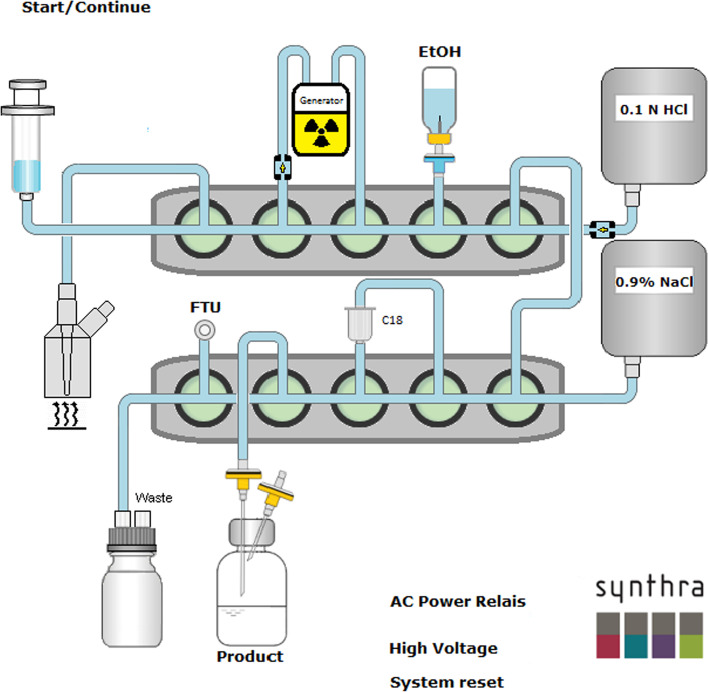
Scheme of the automated GaSy synthesizer from Synthra for [^68^Ga]Ga-FAPI-46 production

For the fully automated synthesis of [^68^Ga]Ga-FAPI-46 using a Synthra synthesizer (operated by the SynthraView Version 5.07.039 software (Synthra, Hamburg, Germany)) shown in Fig. 3 we used a single-use cassette and reagents supplied by Synthra (Synthra, Hamburg, Germany) and ABX. The reagent kit contains HEPES buffer, saline and ethanol vials. In addition, one C18 cartridge is included in the kit which has to be preconditioned prior to use with 2 ml of ethanol and 10 ml of water and dried with air.

Prior to elution of the generator, the reaction mixture containing 5 ml of HEPES buffer solution (5 M), 0.3 mg of ascorbic acid and 50 μg of FAPI-46 were transferred to the reactor vial. The [^68^Ga]GaCl_3_ solution was then eluted from a ^68^Ge/^68^ Ga generator (Eckert & Ziegler, GalliaPharm generator, 1.85 GBq/50 mCi) with 5 ml 0.1 M HCl as described above. The synthesis of [^68^Ga]Ga-FAPI-46 was performed by mixing 50 µg of the FAPI-46 precursor with the [^68^Ga]GaCl_3_ solution directly eluted from the generator at 90 °C and continued heating for 4 min.

For purification of the crude product, 3 ml of isotonic saline were added and passed through a C18 Sep-Pak cartridge into the waste bottle. During this process the [^68^Ga]Ga-FAPI-46 was adsorbed by the cartridge while the ^68^ Ga impurities and HEPES buffer present in the reaction mixture were removed. The C18 cartridge was then flushed with 10 ml of 0.9% aq. NaCl to ensure removal of most of the possibly remaining free [^68^Ga]GaCl_3_ and HEPES buffer and dried with 10 ml of air at the end. The desired [^68^Ga]Ga-FAPI-46 was eluted from the C18 cartridge by flushing it with 3 ml of ethanol and passing the eluate through a 0.22 μm filter to the product vial before being further diluted with 10 ml of 0.9% aq. NaCl.

### Synthesis of [^68^Ga]Ga-FAPI-46 using a Scintomics synthesizer

**Fig. 4:**
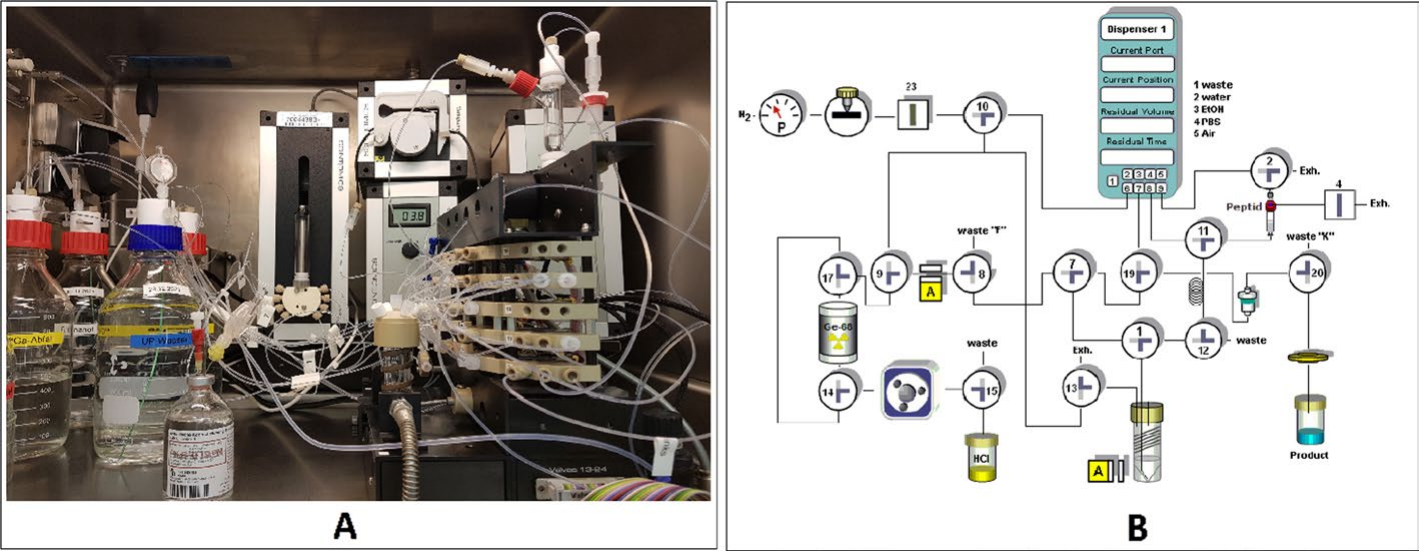
A. Setup for the production of [^68^Ga]Ga-FAPI-46 on GallElut Scintomics synthesizer, and (B) Scheme of the synthesis module

The GallElut Scintomics module (operated by the software Scintomics™ (Scintomics GmbH, Fürstenfeldbruck, Germany)) shown in Fig. 4 was also used for the automated synthesis of [^68^Ga]Ga-FAPI-46 with a customized synthesis system. Prior to the synthesis, the module was cleaned by two automated methods. Firstly, washing with water removed water-soluble contaminants from the peptide vial and the reaction vessel. Secondly, washing with an organic solvent (ethanol) was done to rinse all valves, the peptide vial and the reaction vessel. Finally, all module components were dried using nitrogen gas flow.

HEPES (99.5% purity) was purchased from Sigma-Aldrich. Isotonic saline solution 0.9% was provided by Berlin-Chemie AG (Berlin, Germany). Ultrapure hydrochloric acid (HCl, 30%) and ultrapure water were purchased from Merck Millipore (Merck Millipore, Darmstadt, Germany) and absolute ethanol for analysis was obtained from Carl Roth. The Sep-Pak C18 plus cartridges were purchased from Waters and conditioned with ethanol (10 ml) and water (10 ml) prior to use. Reagents and solvents were added into a glass vial mounted or connected directly on their respective valves. The reaction vessel was equipped with a temperature control device and a radio activity detector.

For the synthesis the glass reactor was first heated to 90 °C. Then, [^68^Ga]GaCl_3_ was eluted from a ^68^Ge/^68^ Ga generator (iThemba generator, 1.85 GBq/50 mCi Somerset West, South Africa) with 2 ml of 1 M HCl into the reaction vial (Fig. 4a). Afterwards 2 ml of HEPES buffer solution containing 0.3 mg of ascorbic acid and 50 μg of FAPI-46 were added into the glass reactor vial. After 4 min at 90 °C, the reaction mixture was cooled down by adding 4.0 mL of 0.9% aq. NaCl and passed through a C18 plus Sep-Pak cartridge and further flushed with 10 mL of 0.9% aq. NaCl. Thereby, the [^68^Ga]Ga-FAPI-46 was adsorbed on the cartridge while the ^68^ Ga impurities and HEPES buffer and traces of ^68^Ge breakthrough present in the reaction mixture were removed. Subsequently, the desired [^68^Ga]Ga-FAPI-46 was eluted from the cartridge by flushing the cartridge with 2 ml of a 1/1 ethanol/water mixture and passed through a 0.22 μm filter to the product vial before being further diluted with 10 mL of 0.9% aq. NaCl.

### [^68^Ga]Ga-FAPI-46 quality control

Quality control (QC) procedures for [^68^Ga]Ga-FAPI-46, based upon the current requirements for radiopharmaceuticals laid out in the European pharmacopoeia for preparation of radiopharmaceuticals, quality control release criteria, are summarized below (Table 1).

**Table 1:** Summary of the product specifications for 50 μg FAPI-46, n = 3

QC Test	Release Criteria	Trasis (n = 3)	Scintomics (n = 3)	Synthra (n = 3)
Yield %	N/A	92.45%	93.32%	92.86%
Visual Inspection	Clear, colorless	Clear, colorless	Clear, colorless	Clear, colorless
Radio chemical identity	RRT^*^ = 0.9–1.1	1.01	1.01	1.01
Radio chemical purity	> 95%	> 99.4%	> 99.7%	> 99.8%
Dose pH	4.0–8.0	6.5	7.5	7.0
Sterile Filter Integrity Test	> 3.2 bar	> 3.2 bar	> 3.2 bar	> 3.2 bar
Radionuclidic Identity (t_1/2_)	62–74	67	67	67
Radionuclide impurity	< 0.001%	0.0004%	0.0001%	0.0001%
Endotoxin analysis	≤ 175 EU/ml	< 5.0 EU/ml	< 5.0 EU/ml	< 5.0 EU/ml
Sterility Testing	No colony growth out to 14 days	pass	pass	pass

Legend: *: relative retention time

### Radio chemical identity und purity

HPLC analysis of radio chemical identity (Table 1) was conducted using a System Gold HPLC System (Beckman Coulter, USA) equipped with an UV detector set to 220 nm and HERM LB 500 γ-detector (Berthold Technologies GmbH & Co. KG, Germany). As the stationary phase a Chromolith HR RP-18 100 × 3 mm HPLC-column (Merck KGaA, Germany) was used. As the mobile phase a gradient of solvents A (H_2_O containing0.1% TFA) and B (acetonitrile containing 0.1% TFA); was used (0–8 min 0–100% B; flow rate: 1.0 mL/min).

The retention time of [^68^Ga]Ga-FAPI-46 was compared to that of the non-active [^nat^Ga]Ga-FAPI-46 reference standard and had to be within ± 10% of the standard relative retention time (RRT).

### TLC analysis

The thin layer chromatography analysis (TLC) of radio chemical purity was conducted using an Elysia-raytest linear analyser detector RITA (Elysia-raytest GmbH, Germany).

- Stationary phase A: Tec-Control blue, (Biodex Medical Systems, USA); mobile phase A: 0.1 M aq. sodium citrate.
- Stationary phase B: iTLC-SG (Agilent Technologies Inc; USA); mobile phase B: 1/1 mixture of 0.4 M aq. ammonium acetate/ethanol).

Radio chemical purity had to be > 95%. All doses met these specifications (Table 1).

### Radionuclidic identity and purity:

The radionuclidic identity and purity were determined by gamma spectroscopy using a High Purity Germanium (HPGe) radiation detector (Ortec, Oak Ridge, TN, USA).

For the determination of the radionuclide identity 10 µl sample of 1:50 dilution the product (5–30 kBq) was analysed by the gamma spectrometer. The measured gamma photons must have energies of 0.511 MeV and 1.077 MeV. Afterward, the half-life was determined by making three measurements of the same sample under the same geometric conditions with a time interval of 15 min. The calculated half-life must be 62 min to 74 min.

For determining the radionuclide purity the same sample was measured after 48 in the gamma spectrometer (measuring time 3 h) under the same geometric conditions for determining the activity of ^68^Ga (resulting from ^68^Ge) and other radionuclidic impurities with a half-life longer than 5 h. The activity after 48 h must be < 0.001% of the eluted activity from the generator.

### Sterile filter integrity test:

The sterile filter (with needle still attached) was connected to a nitrogen supply via a regulator. The needle was submerged in water and the nitrogen pressure was gradually increased. If the pressure was raised above the filter acceptance pressure (3.2 bars) without seeing a stream of bubbles, the filter was considered intact (Table 1).

### Dose pH

The pH of a a small amount of the [^68^Ga]Ga-FAPI-46 solution was determined using Macherey–Nagel® pH 2.0–9.0 non-bleeding pH-indicator strips by visual comparison to the scale provided. As listed in Table 1, the pH was ranging between 6.5 and 7.5.

### Endotoxin analysis

The endotoxin content in the synthetic samples of [^68^Ga]Ga-FAPI-46 was analyzed using a Endosafe® nexgen-PTS™, Charles River. Doses had to contain ≤ 175 Endotoxin Units (EU) / mL to be deemed acceptable. Limulus-amoebocyte-lysate (LAL) test for bacterial endotoxin resulted < 17.5 EU/ml in all of the synthetic samples (Table 1).

### Sterility testing

Fluid thioglycolate media (FTM) plates and soybean casein digest agar media (SCDM) plates were treated with samples of [^68^Ga]Ga-FAPI-46. FTM plates were used to test for anaerobes, aerobes and micro aerophiles whilst SCDM plates were used to test for non-fastidious and fastidious microorganisms. [^68^Ga]Ga-FAPI-46 treated plates were incubated along with positive and negative controls for 14 days. FTM plates were incubated at 32 °C and SCDM plates were incubated at 22 °C according to the current USP guidelines. Plates were visually inspected on the 3rd, the 8th and the 14th day of the test and compared to the positive and negative standards. Positive standards had to show growth (turbidity) on the plates and [^68^Ga]Ga-FAPI-46 negative controls had to have no culture growth after 14 days to be indicative of sterility. All samples met the sterility specifications (Table 1).

### Determination of HEPES

The European Pharmacopoeia prescribes a strict limit of 200 μg per dose for the HEPES content in radiopharmaceuticals intended for intravenous administration.

The HEPES content was determined according to the standard procedure listed in the Ph. Eur. Monograph. As a reference solution 10 mg of HEPES was dissolved in 10 mL of water. 1 mL of this solution was then diluted to 50 mL with water to obtain 20 μg/ml of HEPES.

Two separate spots of reference solution (5 µL) and test solution (a sample of the final product [^68^Ga]Ga-FAPI-46) were put on TLC silica gel F254 plate on aluminum foil (3 cm × 10 cm) and dried in a stream of air. Once the spots were not visible anymore, the TLC plate was developed using a solution of water/acetonitrile (1/3 v/v) as the mobile phase. The development was stopped when the front of the mobile phase reached 2/3 of the plate.

HEPES was visualized by treatment of the dried developed TLC plate by exposure to iodine vapor for 5 min and any spot had not to be more intense than the reference solution, consequently containing only an equal or lower than 20 μg/ml of HEPES.

## Results

**Fig. 5:**
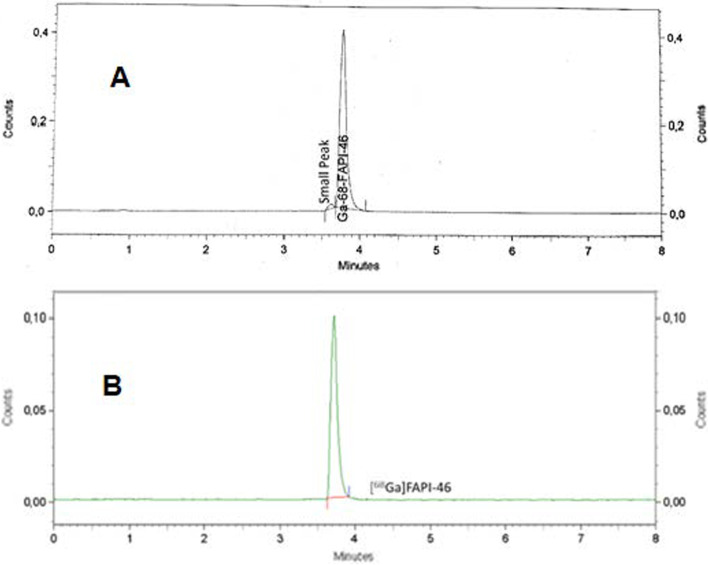
Radio chemical purity detection of [^68^Ga]Ga-FAPI-46 analized via HPLC with UV detector at 220 nm and radioactivity detector (NaI) **A**: for [^68^Ga]Ga-FAPI-46 synthesized using the same cassette and chemistry kit for labelling [^68^Ga]Ga-PSMA-11 (95 °C and 10 min, no addition of ascorbic acid); **B**: the same as A, but with addition of 300 µg of ascorbic acid into the buffer systems.

The analyses of the radiochemical purity of [^68^Ga]Ga-FAPI-46 synthesized with the Synthra and Trasis modules and with the Scintomics system using the same chemistry kits and cassette normally used for DOTATATE labeling with ^68^ Ga showed in all cases an insufficient radiochemical purity of < 95% (Table 2 and Fig. 5A). As shown in the representative radio HPLC chromatogram in Fig. 5A, a poorly separated shoulder with about 5% of the total radioactivity appears next to the main peak at 3.75 min of [^68^Ga]Ga-FAPI-46, regardless of the used synthesis module or generator used.

**Table 2:** Radiochemical purity and yield of [^68^Ga]Ga-FAPI-46

Modules	Generator	Buffer	Radio chemical purity	Radio chemical yield
Trasis	GalliaPharm	Acetate	93.4%	84%
Scintomics	iThemba	HEPES	90.9%	82%
Synthra	GalliaPharm	HEPES	93.2%	84%

Legend: Syntheses conducted using the cassette and chemistry kit for labeling PSMA-11 with ^68^ Ga at 95 °C for 10 min (n = 3).

**Fig. 6:**
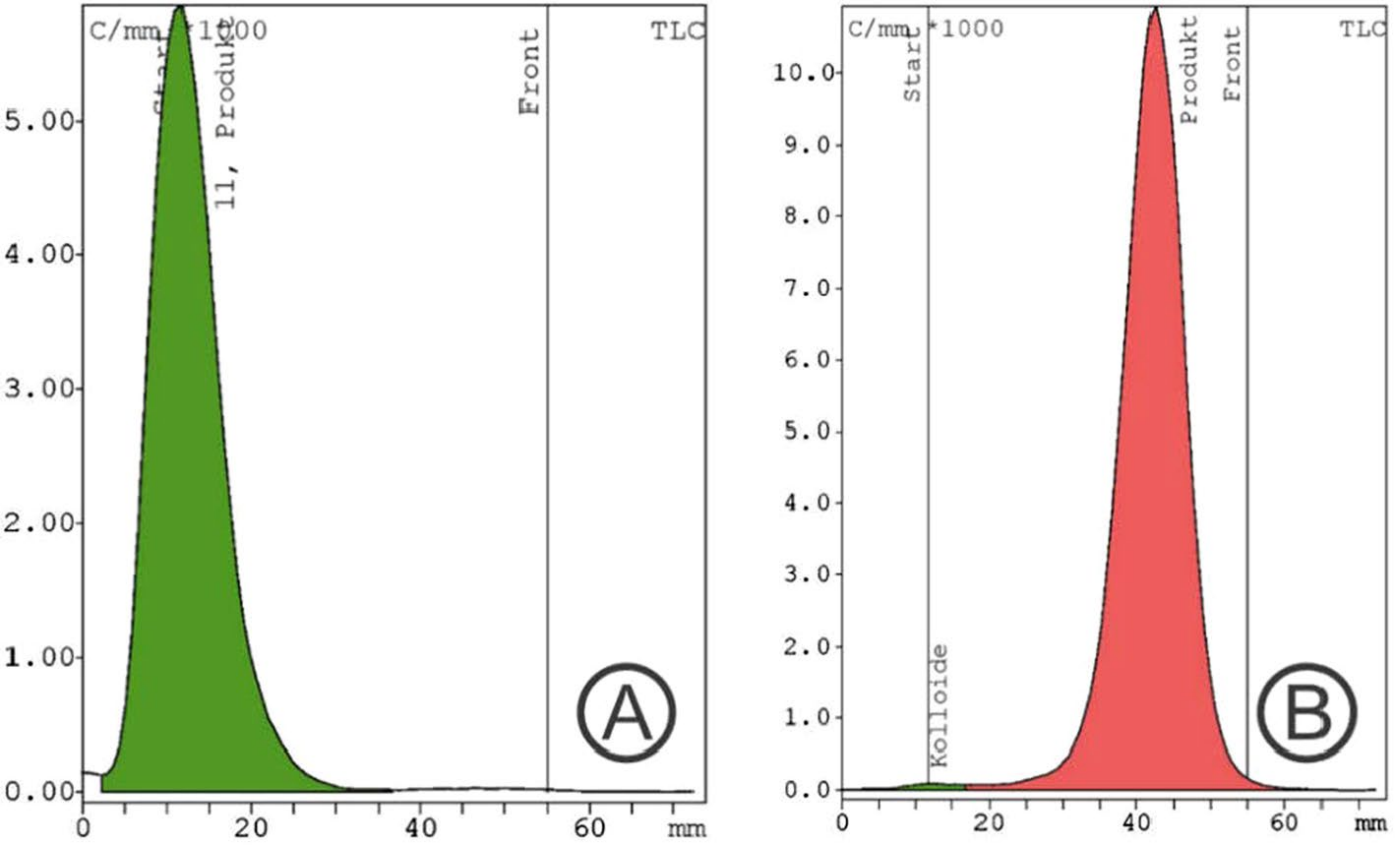
Radiochemical purity analyzed with radio TLC scanner: **A** Stationary phase Tec-Control blue, (Biodex Medical Systems, USA); mobile phase: 0.1 M aq. sodium citrate. **B** Stationary phase iTLC-SG (Agilent Technologies Inc; USA); mobile phase: 1/1 mixture of 0.4 M aq. ammonium acetate/ethanol

In the subsequent experiments, the addition of 300 µg ascorbic acid into the buffered medium bevor starting the labeling led to significant improvement of the radio chemical purity of [^68^Ga]Ga-FAPI-46 to over 99.5% (average of three independent synthesis) as illustrated in a representative radio HPLC chromatogram in Fig. 5b and in the TLC analysis displayed in Fig. 6. Repeated analyzes up to 3 h after synthesis showed that the radio chemical purity was still > 95%.

**Fig. 7:**
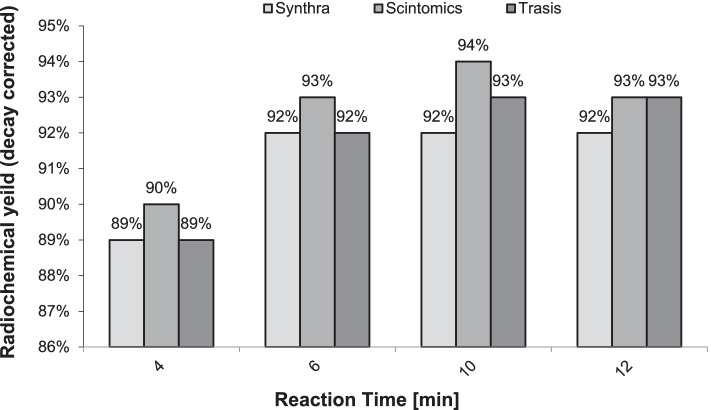
Radiochemical yield of [^68^Ga]Ga-FAPI-46 (n = 3, corrected for decay) versus varied reaction time at 95 °C and 50 µg of FAPI-46 in a buffered medium containing 300 µg ascorbic acid

**Fig. 8:**
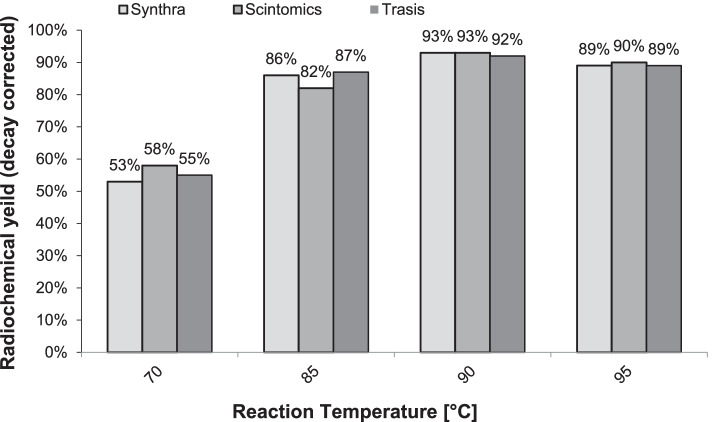
Radio chemical yield of [^68^Ga]Ga-FAPI-46 (n = 3, corrected for decay) versus varied temperature for 4-min reaction time and in buffered medium containing 300 µg ascorbic acid

The studies of the effect of the reaction time and reaction temperature show that the maximum radiochemical yield is reached after 4 min as illustrated in Fig. 7 and the optimal reaction temperature was 95 °C (Fig. 8).

## Discussion

In recent years ^68^ Ga labeled FAPI showed superior diagnosis efficiency in 30 different types of cancer with better tumor background ratio (TBR) comparing with ^18^F-FDG (Fang et al. 2016; Varasteh et al. 2019; Kratochwil et al. 2019). Until early 2020, the most synthesis of [^68^Ga]Ga-FAPI-46 had been performed manually. Then, Spreckelmeyer reported on a fully automated synthesis of [^68^Ga]Ga-FAPI-46 and clinical applications using the commercially available Modular Lab PharmTracer and ML eazy modules with disposable single-use cassettes (Spreckelmeyer et al. 2020).

This is in line with the general transition from manual radiochemistry to the use of automated synthesis modules supported by sophisticated software programs observed in the last couple of decades. These automated systems have clear advantages over manual methods. The fully automated radio labeling processes reduces the exposure of the operator to radioactivity compared to manual methods due to the absence of direct intervention. Furthermore, the automatic synthesis enables the reaction to be performed faster under precisely controlled and reproducible conditions.

However, ^68^ Ga generators have also improved recently in terms of lower ^68^Ge breakthrough and low metal impurity contents.

In our clinic, 1.85 GBq/50 mCi iThemba ^68^Ge/^68^ Ga generators has been used to produce ^68^ Ga-PSMA-11 and ^68^ Ga-DOTATATE without pre-purification since more than ten years. In addition, we have also been using the marketing authorized GalliPharm generator for 2 years. The marketing authorization of the GalliPharm generator guarantees that the European Pharmacopoeia limit for the ^68^Ge breakthrough of < 1 × 10^–3^% is guaranteed over the period of one year. For the iThemba generator which does not have marketing authorization, the periodic check of the ^68^Ge breakthrough is obligatory according to the European Pharmacopoeia regulations. In the case of ^68^Ge breakthrough higher than 1 × 10^–3^% the generator must no longer be used in order to avoid harm to the patients. However, our measurements of the ^68^Ge breakthrough of the iThemba generators have shown a ^68^Ge contamination of < 1 × 10^–3^% in the first nine month of use.

This offers the chance to omit pre-purification steps during the preparation of ^68^ Ga peptides syntheses in general, and hence, it seemed highly attractive to develop GMP-compatible, automated syntheses on a variety of commercial or customized automated systems.

In our clinic, three different synthesis modules are available: the commercial modules Easy One from Trasis and the GaSy from Synthra and the customized Gall Elut synthesis module from Scintomics with fixed tubing.

Our first aim was to synthesize [^68^Ga]Ga-FAPI-46 using the same synthesizer modules, reagents and cartridges which are used for DOTATATE labeling with ^68^ Ga. In both cases, involving the Synthra and the Trasis modules, the same cassette and chemistry kit for labeling with ^68^ Ga was used without modification. In the case of the Scintomics system, the same reagents and cartridge were used as for labeling DOTATATE with ^68^ Ga.

First, we performed three independent experiments for the syntheses of [^68^Ga]Ga-FAPI-46—one with each of the three synthesis modules by mixing 50 μg of FAPI-46 diluted in HEPES or acetate buffer with [^68^Ga]GaCl_3_ directly eluted from the ^68^Ge/^68^ Ga generator. Each complexation was carried out at 98 °C for 10 min. Unfortunately, all of these initial syntheses showed only insufficient radio chemical purity of < 95% (Table 2). A representative HPLC-radio chromatogram is illustrated in Fig. 5A. This chromatogram shows a main peak corresponding to [^68^Ga]Ga-FAPI-46 with a retention time of 3.75 min accompanied by a small shoulder before this main peak. This poorly separated shoulder, which is assumed to result from radiolysis, was about 5% of total radio activity regardless of the used synthesis module or generator.

Da Pieve et al. suggested to add 1 mg of ascorbic acid to the final product to overcome radiolysis problems. Their results showed a radio chemical purity of the product of more than 98% even after 40 min at room temperature with this additive while it decreased to 93.75% in the absence of ascorbic acid (Da Pieve et al. 2022; Mu et al. 2013).

Based on this finding, we added, bevor starting the labeling, 300 µg ascorbic acid into the buffered medium containing 50 µg FAPI-46 in our subsequent experiments. This resulted in a significant improvement of the radio chemical purity of [^68^Ga]Ga-FAPI-46 to over 99.5% (average of three independent synthesis) as also illustrated in a representative chromatogram presented in Fig. 5B that shows only the main peak for [^68^Ga]Ga-FAPI-46 at the expected retention time of 3,75 min, whereas the small shoulder was no longer detectable even after 60 min at room temperature. Even after 3 h the radio chemical purity was still > 95%. Independently, also the TLC analysis confirmed the high radio chemical purity as displayed in Fig. 6.

The short half-life of ^68^ Ga is both an advantage and a challenge. Therefore, radio labeling with such a short half‐lives radio nuclide has to be as efficient and as fast as possible with high labeling yield and high radio chemical purity. In this regard, our second aim was to reduce the reaction time at the appropriate temperature. For this reason, we turned our attention to study the effect of reaction time and temperature. First, we investigated the effect of reaction time at 95 °C. In general, the synthesis is usually considered to be complete when the maximum possible amount of radio activity is incorporated into the tracer molecule. Our experiments showed this maximum to be reached already after 4 min (Fig. 7).

The effect of the reaction temperature was then examined by carrying out the synthesis for 4 min at 70, 85, 90 and 95 °C, respectively. The results are illustrated in Fig. 8 and they clearly show that a temperature of about 90 °C is optimum to achieve the best radiochemical yield.

In summary, the data in Fig. 7 and Fig. 8 revealed a maximum radio chemical yield of [^68^Ga]Ga-FAPI-46 of 92.8 ± 0.5% (average of three independent syntheses, corrected for decay) to be obtained at a temperature of 90 °C and a reaction time of 4 min, respectively, regardless of the choice of the synthesis modules or the generator (Table 3).

**Table 3:** Radiochemical purity and yield of [^68^Ga]Ga-FAPI-46

Modules	Generator	Buffer	Radio chemical purity	Radio chemical yield
Trasis	GalliaPharm	Acetate	99.4%	92.45%
Scintomics	iThemba	HEPES	99.7%	93.3%
Synthra	GalliaPharm	HEPES	99.8%	92.86%

Legend: Syntheses conducted using the cassette and chemistry kit for labeling PSMA-11 with.^68^ Ga at 90 °C and 4 min with 300 µg Ascorbic acid added to the buffer system (n = 3)

A radio chemical yield of 92.8 ± 0.5% compares very well with the yield of 96.0 ± 0.6% reported by Spreckelmeyer et al. (Spreckelmeyer et al. 2020) which needed a tedious pre-purification of the ^68^ Ga eluate from the TiO_2_–based ^68^Ge/^68^ Ga generator by cation exchange to remove the metallic and [^68^Ge]Germanium impurities to reach this level of radio chemical purity and yield. Thus, our approach greatly improves the synthesis because the pre-purification is not necessary anymore. No matter, if the [^68^Ga]Ga-FAPI-46 product was synthesized with ^68^ Ga from a Eckert &Ziegler generator or a ^68^Ge/^68^ Ga iThemba generator the levels of [^68^Ge]Germanium breakthroughs were detected to be < 0.0001% of the total radio activity.

Moreover, careful validation of our syntheses in terms of quality control, like pH, appearance, radio chemical purity, sterility and bacterial endotoxin was in accordance with the European Pharmacopoeia regulations (Table 1). Moreover, the radiochemical purities of all products remained > 95% stability over a 3 h period of incubation at room temperature independent of the commercial or customized automated synthesis module employed.

## Conclusion

Within this study we were able to develop fully automated protocols for the synthesis of [^68^Ga]Ga-FAPI-46 according to GMP standards without the necessity of pre-purification of the ^68^ Ga eluate using three commercial or customized synthesis modules. These protocols allow the synthesis of the labelled target compound within 12 min including process, purification and formulation times with high labeling yield as well as radio chemical and radionuclide purity. Both, the use of an Eckert & Ziegler ^68^Ge/^68^ Ga generator or an iThemba generator led to the same results regarding radiochemical and radionuclide purity with high radiochemical yields.

Thus, we could successfully demonstrate that the synthesis [^68^Ga]Ga-FAPI-46 is practicable on commercial cassette-based systems as well as on customized modules with fixed tubing systems with comparable results. Thereby, the addition of ascorbic acid to the buffer systems was found to be the key to achieve high radiochemical yield and purity. was significantly shortened and simplified. Thus, our optimized and easy to handle protocol significantly shortens and simplifies the synthesis of [^68^Ga]Ga-FAPI-46 making it much more accessible to a broader clinical and scientific community.

## Data Availability

All data generated or analyzed during this study are included in this published article.

## Abbreviations

DPP4: Dipeptidyl peptidase 4
FAPIs: Fibroblast activation protein inhibitors
FAPI: Fibroblast activation protein inhibitor
FAP: Fibroblast activation protein
FTM: Fluid thioglycolate media
GMP: Good manufacturing practice
HEPES: 2-[4-(2-Hydroxyethyl)piperazin-1-yl] ethanesulfonic acid
HLB: Hydrophilic-lipophilic balance
HPLC: High performance liquid chromatography
LAL: Limulus-amoebocyte-lysate
MLPT: Modular lab pharmtracer
ML eazy: Modular lab eazy
PET: Positron emission tomography
Ph Eur: European pharmacopeia
QC: Qquality control
QMA: Quaternary methyl ammonium
TBR: Tumor background ratio
TLC: Thin-layer chromatography
